# Levels and predictors of nurses’ knowledge about diabetes care and management: disparity between perceived and actual knowledge

**DOI:** 10.1186/s12912-023-01504-5

**Published:** 2023-09-28

**Authors:** Bander Albagawi, Sameer A. Alkubati, Rashad Abdul-Ghani

**Affiliations:** 1 Department of Medical Surgical Nursing, College of Nursing, University of Hail, Hail City, Saudi Arabia; 2 Department of Nursing, Faculty of Medicine and Health Sciences, Hodeida University, Hodeida, Yemen; 3 Faculty of Medicine and Health Sciences, Sana’a University, Sana’a, Yemen; 4 Tropical Disease Research Center, Faculty of Medicine and Health Sciences, University of Science and Technology, Sana’a, Yemen

**Keywords:** Diabetes, Perceived knowledge, Actual knowledge, Nurse, Saudi Arabia

## Abstract

**Background:**

Nurses have a crucial role in managing, educating, and caring for diabetic patients. However, their knowledge should be regularly assessed to avoid preventable complications and reduce costs. Therefore, the present study assessed the perceived and actual knowledge about diabetes among nurses in Hail province of Saudi Arabia and investigated predictors of such knowledge.

**Methods:**

A cross-sectional study was conducted among 325 conveniently sampled nurses from all public hospitals and primary healthcare centres in Hail province from September to December 2022. A pre-designed questionnaire was used to collect demographic and practice-related characteristics of the nurses. In addition, data on nurses’ perceived and actual knowledge about diabetes were collected using the Diabetes Self-Report Tool (DSRT) and Diabetes Basic Knowledge Tool (DBKT) self-report questionnaires, respectively. The mean knowledge scores for demographic and practice-related variables were compared using the independent-samples t-test and one-way analysis of variance. Multiple linear regression was used to identify significant predictors of perceived and actual knowledge. The correlation between perceived and actual knowledge was investigated using Pearson’s correlation coefficient. A *P*-value ˂0.05 was considered statistically significant.

**Results:**

Based on a highest maximum score of 60 using the DSRT, the mean score of perceived knowledge was 38.4 ± 12.0, corresponding to a percentage mean score of 64%. On the other hand, based on a highest maximum score of 49 using the DBKT, the mean score of actual knowledge was 23.2 ± 9.6, corresponding to a percentage mean score of 47.3% of correct responses. Being Indian, having a diploma or a bachelor’s degree, and having a poor or fair self-perception of competence in diabetes care were predictors of lower perceived knowledge scores, whereas having no access to diabetes guidelines was a predictor of higher scores. However, being non-Saudi and having experience of at least 16 years were predictors of higher actual knowledge scores. The correlation between actual and perceived knowledge about diabetes was negligible and statistically non-significant (*r* = 0.011, *P* = 0.055).

**Conclusion:**

Nurses affiliated with public health facilities in Hail province lack adequate knowledge about diabetes, with no correlation between what is perceived to be known and what is actually known. Indian citizenship, having a diploma or bachelor’s degree, not having access to diabetes guidelines, not attending courses/workshops, and having a poor or fair self-perception of competence in diabetes care can significantly predict nurses’ perceived knowledge. However, being non-Saudi (Filipino or Indian) and having at least 16 years of experience can significantly predict their actual knowledge of diabetes.

## Introduction

Diabetes mellitus, or diabetes, is a chronic disorder characterised by hyperglycaemia and associated with life-threatening complications, including failure of various organs and systems [1]. The International Diabetes Federation (IDF) estimated that 451 million people had diabetes worldwide in 2017 [2]. Nevertheless, IDF projections estimate the global diabetic population at 693 million people by 2045 [2]. Diabetes is one of the largest epidemics and potential concerns in the twenty-first century due to its rapid global spread, adverse health effects and negative well-being consequences [3]. Diabetes incidence can be influenced by national economic conditions such as limited food resources with high carbohydrate diets and lack of physical education, being more likely to occur in low-income countries [4]. Because of the rapid economic and development progress, as well as changes in nutrition and lifestyle, Middle Eastern countries are expected to be more susceptible to diabetes than other parts of the world. Diabetes poses a huge economic burden on individuals, health systems and countries worldwide. In 2017, it was estimated that $850 billion was spent globally on diabetic patients [2].

Diabetic patients face several compliance issues, including regular monitoring of blood sugar, maintaining it within a normal range, and adhering to treatment schedules [5, 6]. As a result, public awareness campaigns are crucial to promoting people’s understanding of their condition and how to manage it. Education plays an important role in promoting how people take control of their diabetes and improve their quality of life [6]. Nurses who have correct knowledge about diabetes can contribute significantly to educating diabetic patients with the accurate information they need to manage and care for their condition in healthcare and community settings [7, 8]. This role has been demonstrated in a recent randomised controlled trial [8], which found that educational programs for diabetic patients delivered by a primary care nurse over six months, with support sessions after one year, reduced the levels of haemoglobin A1c, blood glucose, blood pressure, low-density lipoprotein, and total cholesterol. It has been proposed that commissioning nurses with a more independent role in diabetes care can boost people’s awareness and their adherence to healthcare regimens [8, 9]. Therefore, nurses must be equipped with in-depth knowledge of diabetes care and management and regularly assessed for such knowledge to provide appropriate counselling and education for diabetic patients. To prepare nurses for the care and management of diabetes, it is essential to equip undergraduate nursing curricula with adequate knowledge and practice and to assess nurses regularly to advance the quality of diabetes care and management based on evidence [10, 11].

Knowledge deficits in various aspects of diabetes care and management have been demonstrated for nurses working in different healthcare settings in Saudi Arabia and elsewhere [12–17]. For instance, a study showed that the perceived knowledge of Swedish nurses about caring for old patients with diabetes was not optimal, lacking the ability to distinguish diabetes types or symptoms [13]. In Saudi Arabia, a gap was found between the perceived and actual knowledge of diabetes among nurses in a tertiary care hospital, with mean scores being 46.9 (of a maximum of 60) for self-reported knowledge and 25.4 (of a maximum of 49 for actual knowledge about diabetes [16]. On the other hand, nurses in Jordan and the United States were found to have insufficient knowledge about diabetes medications [12, 14]. Several factors can influence the levels of knowledge about diabetes among nurses, including demographic and practice-related factors. These factors need to be uncovered to tailor appropriate interventions at individual and institutional levels to increase the quality of diabetes care and management provided by nurses,

In Saudi Arabia, the incidence and prevalence of diabetes are rising alarmingly [16, 18, 19], ranking the second highest in the Middle East and the seventh highest globally [20]. This rise can be attributed to the rising rates of overweight/obesity, smoking, fast food consumption and sedentary lifestyle [21–25]. Diabetes was found in 14.8% of males and 11.7% of females in a 2013 household survey [24]. According to the estimates of IDF in 2021 [26], diabetes is prevalent among 17.7% of adults in the country, and the diabetes-related expenditure per a diabetic adult is $1,745 on average.

With the dramatic rise in diabetes worldwide, including in Saudi Arabia, there is a need for continuing education to improve nursing knowledge about diabetes care and management. This knowledge is critical for effective disease management and prevention of its complications, particularly in primary healthcare (PHC) settings [27]. Besides caring for diabetic patients, nurses play a key role in educating them about diabetes self-care and management with up-to-date information, including dietary recommendations, medication intake and regimens and blood glucose monitoring [16, 28].

In its National Strategic Plan (Vision 2030), the Saudi government places high emphasis on controlling and phasing out chronic illnesses in the country through multi-pronged approaches such as continuous screening, educational interventions and treatment activities [29]. Therefore, Saudi Arabia’s healthcare system is struggling to meet the gold standard of nursing practice due to a lack of nursing professionals and sociocultural concerns [30–32], and the Ministry of Health has also launched an anti-diabetes educational program to expand public awareness and knowledge related to diabetes [33]. Because nurses are often the first members to interact with people during diabetes screening, care and education, health systems rely on them to educate, care for, and manage diabetic patients. Therefore, they play a vital role in a large part of the success of such interventions. Nevertheless, discrepancies between what they perceive to know and what they actually know about diabetes can limit their ability to support diabetic patients.

In Hail province in the northwest of Saudi Arabia, 20% of adults were reported to have diabetes in 2018, which is the second highest prevalence of diabetes in the country [34]. Because nurses have a key role in addressing this issue, there is a need to improve their knowledge and competence in diabetes care and management. The disparity between perceived and actual knowledge may influence nurses’ competence in caring for and managing diabetes. However, nurses’ perceived and actual knowledge about diabetes in Saudi Arabia is largely unknown, particularly in the country’s northwest region. To the best of our knowledge, there have been no published studies investigating the relationship between the perceived and actual knowledge of nurses about diabetes care and management in the northwest region of the country. Therefore, this study assessed perceived *versus* actual knowledge about diabetes among nurses in Hail province, assuming that nurses’ perceived knowledge would be positively correlated with their actual knowledge about diabetes care and management. In addition, the predictors of both types of knowledge were analysed.

## Methods

### Study design and population

This cross-sectional study was conducted among nurses affiliated with four public hospitals and PHC centres in Hail province from September to December 2022. Saudi and non-Saudi nurses providing care and management to diabetic patients attending the departments of the hospitals and PHC centres were included if they gave written informed consent to participate voluntarily. Nurses were excluded if they had less than one year of experience, held only administrative roles, or were working in departments providing support services such as radiology and laboratory departments. A sample size of 292 nurses was determined using OpenEpi web-based calculator, Version 3.01 (www.openepi.com) based on the following criteria: 95% confidence level, 5% absolute precision and a population size of 1200. However, the questionnaire was distributed to 500 nurses; of whom, 325 completed the questionnaire with a response rate of 65%.

### Study tools

The Diabetes Self-Report Tool (DSRT) and Diabetes Basic Knowledge Tool (DBKT) self-report questionnaires were used to collect data on nurses’ knowledge about diabetes [35]. A pre-designed questionnaire was also used to collect nurses’ demographic and practice-related characteristics, including gender, age, citizenship, level of qualification, years of experience, participation in diabetes-related courses/workshops, access to diabetes management guidelines, and perceived competence in the care and management of diabetic patients.

Perceived knowledge about diabetes was assessed using the DSRT, which included 15 items related to the aetiology of types 1 and 2 diabetes, complications, medications, symptom management, blood glucose monitoring, dietary recommendations and diabetic ketoacidosis. This tool measures the nurses’ perceived ability to describe or complete tasks related to diabetes through agreement or disagreement with these items using a four-point Likert scale, where responses were scored from 4 for strongly agree to 1 for strongly disagree. Accordingly, the overall score for all items ranged from 15 to 60, with a higher score denoting greater perceived knowledge. On the other hand, actual knowledge about diabetes was assessed using the DBKT, which included 45 multiple-choice questions related to knowledge about diabetes, including medications, pathology, symptoms, diet, blood glucose monitoring and care for diabetic patients. In addition, four items by Alotaibi et al. [16] addressing knowledge about diabetes complications were added. On scoring each correct answer as 1 and incorrect answer as 0, the overall score for all items ranged between zero and 49, with a higher score denoting greater actual knowledge.

The content validity index of the study tool among Saudi nurses was previously established at 0.98 [16]. In addition, the content validity of study tools was judged by seven experts (two academic nurses, two endocrinologists, and three nurses working in diabetes clinics), where a minor rewording was done according to their suggestions, but nothing else was added or deleted. The tools’ overall content validity index was 0.96. The questionnaires were confirmed reliable by a pilot study among 35 nurses not included in the study sample. Their internal consistency was acceptable, as revealed by Cronbach’s alpha coefficients of 0.896 and 0.893 for DBKT and DSRT, respectively. The questionnaire took approximately 40 min to be completed by nurses.

### Data collection

After the pilot study was completed, the head nurses in the study hospitals and PHC centres encouraged nurses to participate, who were then invited by the researchers. After explaining the purpose of the study, a researcher distributed the questionnaires along with informed consent forms to the nurses during their breaks and waited in the department head’s office until all questionnaires were completed. To maintain privacy and prevent personal information from being identified by anyone other than the researchers, the nurses were asked not to write down their names on the questionnaires, while informed consent forms were completed, signed and received by the researchers as the questionnaires were distributed. The anonymised questionnaires were then collected by one of the nurses and delivered to the department head’s office to facilitate questionnaire collection. In addition, the participants’ anonymity was maintained that only aggregated data were communicated. The participants’ confidentiality was ensured through using a code number during the data collection and analysis for each participant.

### Statistical analysis

Data were analysed using the IBM SPSS Statistics software, Version 27 (IBM Corp., Armonk, NY, USA). Independent-samples *t*-test and one-way analysis of variance (ANOVA) with Scheffé post hoc test, whichever is appropriate, were used to compare the mean knowledge scores for demographic characteristics (gender, age, citizenship, type of facility, level of qualification, and years of experience) and practice-related factors (direct involvement in diabetes care, availability of and adherence to policies and guidelines, participation in courses/workshop, and perceived competence in caring for diabetic patients). Multiple linear regression was used to identify significant predictors of perceived and actual knowledge about diabetes among nurses. Multiple linear regression was then used to identify significant predictors of perceived and actual knowledge about diabetes among nurses. Correlation between perceived and actual knowledge was tested using Pearson’s correlation coefficient. A *P*-value ˂0.05 was considered statistically significant.

## Results

### Characteristics of participating nurses

Table 1 shows that the majority of nurses were females (61.8%) and aged 30 years or younger, with a mean age of 22.6 ± 1.5 (range: 20–62). Saudi nurses represented less than half of the respondents, followed by Filipinos (30.8%) and Indians (20.9%). On the other hand, the majority of nurses had a bachelor’s degree (76.9%), had 5–10 years of experience (39.1%) and were directly involved in diabetes care (79.7%).

**Table 1:** Characteristics of nurses enrolled in the study (N = 325)

Characteristic		*n*	(%)
Gender			
	Male	124	(38.2)
	Female	201	(61.8)
Age (years)			
	$\le$30	172	(52.9)
	> 30	153	(47.1)
	Mean ± SD: 22.6 ± 1.5		
	Range: 20–62		
Citizenship			
	Saudi	157	(48.3)
	Filipino	100	(30.8)
	Indian	68	(20.9)
Type of facility			
	Hospital	267	(82.2)
	PHC centre	58	(17.8)
Level of qualification			
	Diploma	38	(11.7)
	Bachelor’s	250	(76.9)
	Master’s	37	(11.4)
Length of experience (years)			
	< 5	119	(36.6)
	5–10	127	(39.1)
	11–15	48	(14.8)
	≥ 16	31	(9.5)
Direct involvement in diabetes care			
	Yes	259	(79.7)
	No	66	(20.3)

SD, standard deviation; PHC, primary healthcare

### Nurses’ perceived knowledge about diabetes

**Fig. 1:**
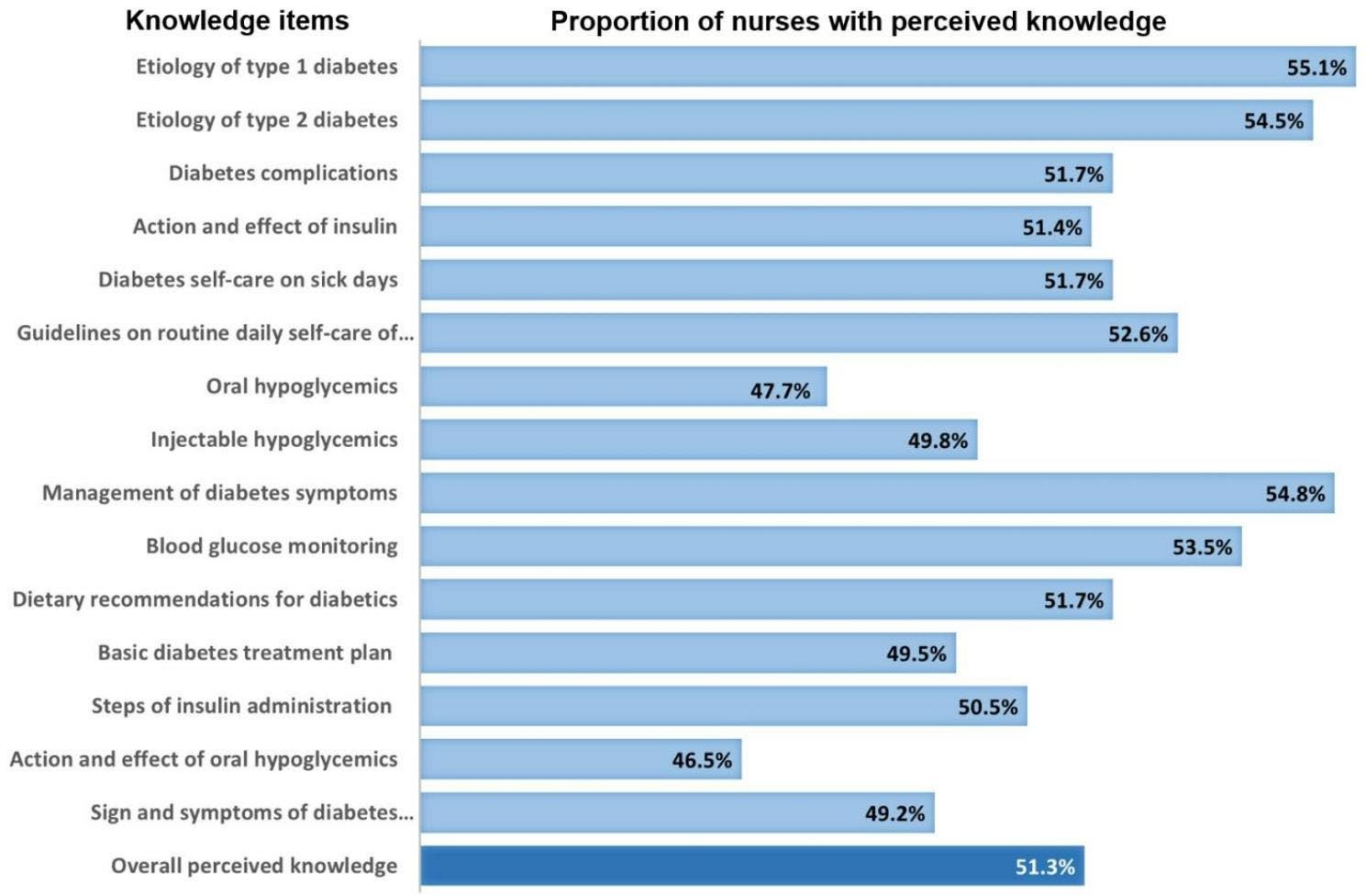
Perceived knowledge of diabetes among nurses

Based on a highest maximum score of 60 using the DSRT, the overall mean score of perceived knowledge was 38.4 ± 12.0, corresponding to a percentage mean score of 64%. Figure 1 shows that the overall proportion of nurses with correct perceived knowledge about diabetes was 51.3%. Correct perceived knowledge about diabetes was reported by more or less than 50% of respondents for each of the 15 knowledge items of the tool, ranging from 46.5% for knowledge about the action and effects of oral hypoglycaemics to 55.1% for knowing the aetiology of type 1 diabetes.

### Nurses’ actual knowledge about diabetes

Based on a highest maximum score of 49 using the DBKT, Table 2 shows that the overall mean score of nurses’ actual knowledge about diabetes care and management was 23.2 ± 9.6, corresponding to 47.3% correct responses. The highest percentage mean scores of correct responses were observed for knowledge about monitoring and complications (56.9%) as well as pathology, symptoms and management (47.1%), followed by a percentage mean score of 45% for knowledge about diabetes medications. However, the lowest percentage mean score was observed for knowledge about diabetes diet/nutrition (33.3%).

**Table 2:** Categories of actual knowledge about diabetes among nurses

Knowledge category	*N*	Mean score ± SD	% Mean score of correct answers
Diabetes pathology, symptoms and management	14	6.6 ± 2.9	47.1
Diabetes monitoring and complications	13	7.4 ± 3.7	56.9
Diabetes diet/nutrition	6	2.0 ± 1.2	33.3
Diabetes medications	16	7.2 ± 3.9	45.0
Overall score	49	23.2 ± 9.6	47.3

N, number of items per category; SD, standard deviation

### Comparison of diabetes-related knowledge scores by demographic characteristics

Table 3 shows that females scored significantly higher than males in perceived (*P* = 0.001) and actual knowledge about diabetes (*P* < 0.001). Nurses older than 30 years (*P* = 0.002) and those affiliated with hospitals (*P* = 0.030) showed significantly higher actual knowledge scores compared to their peers, but perceived knowledge did not differ significantly by age or type of facility. When compared to Saudi and Indian nurses regarding both perceived and actual knowledge about diabetes, Filipino nurses scored significantly higher (*P* < 0.001). Nurses with a master’s degree scored significantly higher in perceived (*P* < 0.001) and actual (*P* = 0.009) knowledge compared to those having a diploma or bachelor’s degree. On the other hand, nurses experienced for ≥ 16 years scored significantly higher in perceived (*P* = 0.004) and actual (*P* = 0.021) knowledge compared to their peers.

**Table 3:** Comparison of diabetes-related knowledge scores of nurses by demographic characteristics

Variable		Perceived knowledge score		Actual knowledge score	
		Mean ± SD	*P-*value	Mean ± SD	*P-*value
Gender					
	Male	45.4 ± 6.3	0.001	18.6 ± 8.2	< 0.001
	Female	51.2 ± 6.9		31.1 ± 7.7	
**Age** (years)					
	≤ 30	46.7 ± 6.9	0.128	22.1 ± 9.8	0.002
	> 30	49.6 ± 7.1		26.3 ± 10.1	
**Citizenship**					
	Saudi	39.9 ± 10.3	< 0.001	17.8 ± 8.2	< 0.001
	Filipino	40.9 ± 13.3		30.9 ± 7.6	
	Indian	31.3 ± 11.0		24.6 ± 6.8	
**Type of facility**					
	Hospital	38.0 ± 12.3	0.166	23.7 ± 9.9	0.030
	PHC centre	40.4 ± 10.4		20.7 ± 7.3	
**Level of qualification**					
	Diploma	29.5 ± 7.6	< 0.001	19.0 ± 9.4	0.009
	Bachelor’s	38.5 ± 10.9		23.6 ± 9.1	
	Master’s	47.2 ± 16.1		25.2 ± 11.8	
**Length of experience** (years)					
	< 5	46.0 ± 6.8	0.004	21.7 ± 9.9	0.021
	5–10	48.4 ± 6.3		23.1 ± 9.3	
	11–15	48.6 ± 7.2		24.5 ± 9.7	
	≥ 16	55.1 ± 7.5		27.4 ± 8.2	

SD, standard deviation

### Comparison of diabetes-related knowledge scores by practice-related factors

Table 4 shows that there was no significant difference in the mean scores of perceived (*P* = 0.067) and actual (*P* = 0.116) diabetes knowledge between nurses who were directly involved in diabetes care and those who were not. Nurses who reported having diabetes management policy in place (*P* = 0.002) and those who had access to diabetes management guidelines (*P* < 0.001) scored significantly higher in perceived knowledge compared to their peers. However, there was no statistically significant difference between the scores of actual knowledge regarding the availability of policy (*P* = 0.803) or access to management guidelines (*P* = 0.063). Nurses who participated in diabetes-related courses/workshops scored significantly higher in perceived knowledge (*P* = 0.031) but significantly lower in actual knowledge about diabetes (*P* < 0.001). On the other hand, neither perceived (*P* = 0.078) nor actual (*P* = 0.077) diabetes knowledge scores differed significantly between nurses who reported providing diabetes care after referring to management guidelines/policies and those who did not. Nurses who perceived having excellent or good competence in caring for diabetic patients scored significantly higher in perceived diabetes knowledge compared to their peers (*P* < 0.001). However, the mean score of actual knowledge did not significantly differ by the nurses’ perceived level of competence (*P* = 0.257).

**Table 4:** Comparison of diabetes-related knowledge scores of nurses by practice-related factors

Variable		Perceived knowledge score		Actual knowledge score	
		Mean ± SD	*P-*value	Mean ± SD	*P-*value
Direct involvement in diabetes care					
	Yes	48.5 ± 7.0	0.067	24.4 ± 10.0	0.116
	No	45.4 ± 7.1		21.2 ± 9.9	
**Availability of diabetes management policy in place**					
	Yes	48.9 ± 6.9	0.002	23.6 ± 11.0	0.803
	No	45.0 ± 7.0		23.9 ± 7.5	
**Access to diabetes management guidelines**					
	Yes	48.9 ± 6.9	< 0.001	24.9 ± 10.9	0.063
	No	45.9 ± 7.1		21.7 ± 8.3	
**Attending diabetes-related courses/workshops**					
	Yes	49.2 ± 6.7	0.031	20.3 ± 10.5	< 0.001
	No	47.1 ± 7.2		25.2 ± 9.5	
**Providing diabetes care after referring to management guidelines/policy**					
	Rarely	22.1 ± 9.9	0.078	40.7 ± 11.1	0.077
	Always	25.1 ± 8.7		34.6 ± 12.5	
**Perceived competence in caring for diabetic patients**					
	Poor	23.6 ± 7.3	< 0.001	22.2 ± 7.7	0.257
	Fair	30.9 ± 7.6		23.4 ± 9.0	
	Good	43.8 ± 8.7		24.0 ± 9.6	
	Excellent	45.1 ± 14.3		21.1 ± 11.3	

SD, standard deviation

### Predictors of knowledge about diabetes among nurses

Multiple linear regression analysis of demographic and practice-related variables as predictors of perceived and actual knowledge scores shows that the model was significant for both types of knowledge (*P* < 0.001). It accounted for 36.9% (*R*^*2*^ = 0.368, adjusted *R*^*2*^ = 0.369) and 50.6% (*R*^*2*^ = 0.524, adjusted *R*^*2*^ = 0.506) of the variance in perceived and actual knowledge, respectively (Table 5).

Compared to reference categories, being Indian (*P* = 0.001), having a diploma or a bachelor’s degree in nursing (*P* < 0.001) and having a poor or fair self-perception of competence in diabetes care (*P* < 0.001) were significant predictors of lower scores of perceived knowledge about diabetes among nurses, whereas lack of access to diabetes guidelines was a significant predictor of higher scores of perceived knowledge. On the other hand, being non-Saudi (Filipino or Indian) (*P* < 0.001) and having experience of at least 16 years (*P* = 0.032) were significant predictors of higher scores of actual knowledge about diabetes among nurses (Table 5).

**Table 5:** Multiple linear regression of predictors of knowledge about diabetes among nurses

Predictors	Perceived knowledge*			Actual Knowledge**		
	β	95% CI for β	*P*-value	β	95% CI for β	*P-*value
Gender						
Male	Reference			Reference		
Female	2.67	0.45–2.23	0.065	1.86	0.50–1.99	0.131
**Age** (years)	NA			-0.03	--0.51–1.97	0.715
**Citizenship**						
Saudi	Reference			Reference		
Filipino	-1.97	-0.04–2.36	0.208	10.73	0.40–2.49	˂0.001
Indian	-5.22	-0.53–1.88	0.001	5.01	0.54–1.85	˂0.001
**Type of facility**						
Hospital	NA			Reference		
PHC centre				-0.97	-0.94–1.07	0.396
**Level of qualification**						
Diploma	-13.92	-0.47–2.12	˂0.001	-1.30	-0.44–2.28	0.513
Bachelor’s	-6.290-	-0.47–2.15	˂0.001	2.33	0.47–2.15	0.115
Master’s	Reference			Reference		
**Length of experience** (years)	-0.01-	-0.65–1.55	0.916	2.15	0.52–1.91	0.032
**Policy availability**						
Yes	Reference			Not applicable		
No	1.43	0.51–1.98	0.366			
**Access to guidelines**						
Yes	Reference			Not applicable		
No	3.27	0.51–1.95	0.031			
**Attending courses/workshops**						
Yes	Reference			Reference		
No	0.049	0.73–1.36	0.965	1.22	0.85–1.18	0.195
**Perceived competence**						
Poor	-19.78	-0.58–1.72	˂0.001	Not applicable		
Fair	-14.06	-0.40–2.52	˂0.001			
Good	-2.11	-0.39–2.55	0.162			
Excellent	Reference					

*R^2^ = 0.386, adjusted *R*^2^ = 0.369, *P* ˂0.001; **R^2^ = 0.524, adjusted *R*^2^ = 0.506, *P* ˂0.001; CI, confidence interval

### Correlation between perceived and actual knowledge about diabetes

**Fig. 2:**
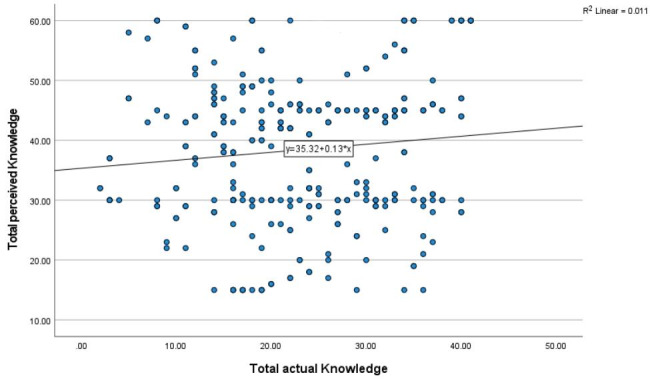
Pearson’s correlation between the levels of nurses’ perceived and actual knowledge

Figure 2 shows a negligible non-significant correlation between the actual and perceived knowledge of nurses about diabetes (*r* = 0.011, *P* = 0.055).

## Discussion

Understanding perceived and actual knowledge about diabetes care and management in nursing practice is critical for developing educational interventions to improve nurses’ professional competence. Nevertheless, and to the best of our knowledge, the relationship between both types of knowledge has not been assessed among nurses in Hail province so far. Such a relationship was negligible in the present study, reflecting the wide gap between what is perceived to be known by the nurses and what they actually know about diabetes. This disparity could be explained by the failure to seek guidelines and education resources by nurses perceiving themselves as knowledgeable [35].

The finding of the present study is consistent with that reported for nurses in a tertiary hospital in Riyadh, Saudi Arabia [16], where the correlation between both types of knowledge was moderate. Therefore, the gap between nurses’ perceptions of their knowledge and their real knowledge needs to be bridged through educational interventions and continuing education programs because it can greatly influence nurses’ competence in caring for and managing diabetic patients. The prevalence and economic burden of diabetes in the country [24, 36] warrant corrective measures to educate nurses about the guidelines and policies for diabetes care and management. In line with the present study, inadequate levels of knowledge about diabetes have been reported among nurses from developed countries, including the United States [35, 37] and the United Kingdom [38, 39].

Inconsistent with the present study, positive correlations were found between actual and perceived levels of knowledge about diabetes among registered nurses in Jordan [14] and the United States [37]. Conversely, Drass et al. [35] reported a low negative correlation between the perceived and actual knowledge of nurses, showing the inverse relationship between both types of knowledge. In another context, no relationship was found between perceived competence and actual knowledge about diabetes among staff nurses in the United States [40].

Approximately two-thirds of nurses in the present study, as shown by a percentage mean score of 64%, had positive attitudes towards their perceived knowledge about diabetes care and management, which is lower than those reported for nurses in Riyadh (78.2%) [16] and Jordan (71.8%) [14]. The perceived knowledge as measured by the DSRT in the present study showed that more or less than half of nurses were knowledgeable about individual items of the tool. The low proportions of self-perceived knowledge about various aspects related to diabetes reveal knowledge deficits in diabetes care and management and underscore the importance of continuing education on diabetes care and management guidelines. Furthermore, this finding was confirmed by the low level of actual knowledge, as shown by the drop in the percentage mean score of correct answers to the questions of the DBKT to 47.3%. A slightly higher percentage mean score of correct answers to the DBKT questions (52.3%) was reported for nurses in Riyadh [16]. In addition, higher percentage mean scores of correct responses have been reported among nurses in Jordan (63.4%) [14], and the United States (64–75%) [35, 40].

In the present study, the significantly higher score of perceived knowledge about diabetes among female nurses was translated into a significantly higher score of actual knowledge, which may be partly attributed to the more interest given by females to update their information. In Riyadh, male nurses scored significantly higher in perceived knowledge but scored significantly lower in actual knowledge about diabetes care and management compared to females [16]. On the other hand, the level of actual, but not perceived, knowledge significantly increased with increasing age, being significantly higher among those aged over 30 years compared to younger ones. This increase could be attributed to the longer years of nursing experience of older nurses, which was evidenced by their significantly higher scores in both perceived and actual knowledge compared to those with fewer years of experience. Moreover, nursing experience for at least 16 years was a significant predictor of higher scores of actual knowledge in the present study. In contrast, actual knowledge about diabetes did not significantly correlate with the experience of nurses in Jordan, the United Kingdom and the United States [14, 38]. Regression analysis failed to reveal gender or age as significant predictors of either type of knowledge in the present study, despite the significant differences in the mean knowledge scores by gender and age of nurses. In contrast, a recent study among nursing students in Riyadh found gender to be a significant predictor of perceived knowledge and age to be a significant predictor of actual knowledge about diabetes [41].

The present study revealed that non-Saudi nurses (Filipinos and Indians) scored significantly higher than Saudi nurses on perceived and actual knowledge about diabetes, with Filipino nurses exhibiting higher levels of knowledge. Similarly, Saudi nurses in Riyadh were found to be less knowledgeable about diabetes compared to expatriate nurses [16]. In the present study, regression analysis showed that Indian citizenship was a significant predictor of lower scores of perceived knowledge compared to Saudi citizenship; however, non-Saudi citizenship (Filipino and Indian) was a significant predictor of higher scores of actual knowledge. For this reason, and among others, it is common for Saudi Arabian organisations to favour expatriate nurses to improve the quality of healthcare provided [31]. Such a difference could be attributed to variations in the calibre of nursing education and training across the countries. This underscores the need to improve nursing education and offer continuing education for staff nurses in the country. Saudi government provides international scholarships to qualify Saudi nurses abroad, mainly in Western countries [42]. The role of the level of qualification in improving nurses’ knowledge about diabetes care and management is evident in the present study, where nurses with a master’s degree scored significantly higher compared to those less qualified. This finding is consistent with that reported for nurses in Riyadh [16]. In another context, a strong positive correlation was reported between the level of education and actual knowledge about diabetes among Jordanian nurses [14].

Engagement in care for diabetic patients can considerably contribute to the acquisition or retention of knowledge about diabetes care [15]. Although nurses caring for diabetic patients are expected to be more knowledgeable about diabetes care compared to their colleagues caring for other patient groups, neither perceived nor actual knowledge has been significantly impacted by the direct involvement in diabetes care in the present study. In particular, knowledge deficits among nurses directly involved in caring for diabetic patients can have serious clinical implications because of the complications that could arise from knowledge issues. In contrast, Alotaibi et al. [16] found significantly higher levels of perceived and actual knowledge among nurses providing diabetes care compared to non-diabetic care providers. Additionally, perceived knowledge was significantly predicted by the experience of nursing students in direct care for diabetic patients in Riyadh [41].

Nurses’ perceived, but not actual, knowledge significantly increased with access to the policies or guidelines of diabetes management among nurses in the present study. However, lack of access to diabetes management guidelines was a significant predictor of lower scores of perceived knowledge in the present study. Further investigation of this issue is necessary to identify the factors related to the lack of benefit from such resources to improve nurses’ actual knowledge. Access to diabetes management policies and guidelines alone is not sufficient but can contribute to strengthening nurses’ knowledge and competence. Therefore, continuing education and regular assessment should be considered to improve nurses’ knowledge and competence. In contrast to the present study, having access to the policies or guidelines of diabetes management by nurses scored significantly higher in perceived and actual knowledge in Riyadh [16].

In the present study, attending diabetes-related courses and training workshops led to a significantly higher mean score of perceived knowledge but a significantly lower score of actual knowledge about diabetes. The content and number of such courses/workshops may influence their impact on the acquisition of actual knowledge. Poor attendance at continuing diabetes education is an obstacle to nurses’ acquisition and retention of knowledge [15]. On the contrary, attending diabetes-related courses/workshops led to significantly higher mean scores of both perceived and actual knowledge about diabetes among nurses in Riyadh [16]. Therefore, it is necessary to identify the need for continuing diabetes education to improve the knowledge and skills of nurses who provide diabetes education, management and care [43]. Problem-based learning and simulations have been suggested as effective educational interventions to improve nurses’ skills in managing chronic diseases [44].

In the present study, nurses’ self-perception of their competence in diabetes care was not a predictor of their actual knowledge. Nevertheless, poor or fair self-perception of competence in diabetes care was a significant predictor of lower scores of perceived knowledge, and nurses who rated their competence as poor or fair also scored significantly lower in perceived knowledge. In contrast, nurses in Riyadh who rated their perceived competence in diabetes care as poor scored significantly higher in both perceived and actual knowledge about diabetes [16]. A study among nurses in the United States found no relationship between perceived competence in diabetes care and actual knowledge [40]. Therefore, there is a need to improve the professional competence of nurses in diabetes care and management because their quality requires the involvement of general practice nurses [15].

To summarise, nurses in Hail province performed better in terms of perceived *versus* actual knowledge about diabetes care and management. This knowledge discrepancy can potentially affect the quality of clinical care and education provided by nurses to diabetic patients in Saudi Arabia’s northwest region. To ensure that nurses provide the best practice possible, it is vital to equip them with the knowledge and skills needed for diabetes care and management throughout their undergraduate studies by enhancing curricula and by regular in-service training during their work. Together with previous findings from other regions of the country, there is a need to build a national framework for diabetes nursing knowledge and competence, considering the demographic and practice-related aspects that help to predict each type of knowledge.

Despite the participation of nurses from all public hospitals and PHC centres in Hail province, the present study has some limitations. First, it assessed perceived knowledge using a self-reported questionnaire, which could overestimate or underestimate nurses’ knowledge scores because of reporting bias. Second, this study adopted a convenience sampling approach from the public sector in a single province of the country, which may limit the generalizability of the results to the private sector and other regions of the country. However, it revealed the existence of a gap between what nurses perceive to know and what they actually know about diabetes care and management in a region with no published data in this respect. Therefore, further nationwide studies are recommended to better assess nurses’ knowledge about diabetes care and management. The time required to answer a large number of questions on the study questionnaires might affect nurses’ response rate.

## Conclusion

Nurses affiliated with public health facilities in Hail province lack adequate knowledge about diabetes, with no correlation between what is perceived to be known by nurses and what they actually know. Indian citizenship, having a diploma or bachelor’s degree, not having access to diabetes guidelines, not attending courses/workshops, and having a poor or fair self-perception of competence in diabetes care can significantly predict nurses’ perceived knowledge. However, being non-Saudi (Filipino or Indian) and having at least 16 years of experience can significantly predict their actual knowledge of diabetes.

## Data Availability

The data presented in this study are available on request from the corresponding author.

## Abbreviations

ANOVA: Analysis of variance
CI: Confidence interval
DSRT: Diabetes Self-Report Tool
DBKT: Diabetes Basic Knowledge Tool
IDF: International Diabetes Federation
PHC: Primary healthcare
SD: Standard deviation
SPSS: Statistical Package for the Social Sciences
